# Cross-reactivity of antibodies from non-hospitalized COVID-19 positive individuals against the native, B.1.351, B.1.617.2, and P.1 SARS-CoV-2 spike proteins

**DOI:** 10.1038/s41598-021-00844-z

**Published:** 2021-11-08

**Authors:** Maryam Hojjat Jodaylami, Abdelhadi Djaïleb, Pierre Ricard, Étienne Lavallée, Stella Cellier-Goetghebeur, Megan-Faye Parker, Julien Coutu, Matthew Stuible, Christian Gervais, Yves Durocher, Florence Desautels, Marie-Pierre Cayer, Marie Joëlle de Grandmont, Samuel Rochette, Danny Brouard, Sylvie Trottier, Denis Boudreau, Joelle N. Pelletier, Jean-Francois Masson

**Affiliations:** 1 Department of Chemistry, Québec Centre for Advanced Materials (QCAM), Regroupement Québécois sur les Matériaux de Pointe (RQMP), and Centre Interdisciplinaire de Recherche sur le Cerveau et l’apprentissage (CIRCA), Université de Montréal, CP 6128 Succ. Centre-Ville, Montréal, QC H3C 3J7 Canada; 2 Department of Chemistry, Department of Biochemistry and PROTEO, The Québec Network for Research On Protein Function, Engineering and Applications, Université de Montréal, CP 6128 Succ. Centre-Ville, Montréal, QC H3C 3J7 Canada; 3 Mammalian Cell Expression, Human Health Therapeutics Research Centre, National Research Council Canada, Montréal, QC Canada; 4 Héma‐Québec, Affaires médicales et innovation, 1070, avenue des Sciences‐de‐la‐Vie, Québec, QC G1V 5C3 Canada; 5 Centre de recherche du Centre hospitalier universitaire de Québec and Département de microbiologie-infectiologie et d’immunologie, Université Laval, 2705, boulevard Laurier, Québec, QC G1V 4G2 Canada; 6 Department of Chemistry and Centre for Optics, Photonics and Lasers (COPL), Université Laval, 1045, av. de la Médecine, Québec, QC G1V 0A6 Canada

**Keywords:** Antibodies, Optical sensors

## Abstract

SARS-CoV-2 variants of concern (VOCs) have emerged worldwide, with implications on the spread of the pandemic. Characterizing the cross-reactivity of antibodies against these VOCs is necessary to understand the humoral response of non-hospitalized individuals previously infected with SARS-CoV-2, a population that remains understudied. Thirty-two SARS-CoV-2-positive (PCR-confirmed) and non-hospitalized Canadian adults were enrolled 14–21 days post-diagnosis in 2020, before the emergence of the B.1.351 (also known as Beta), B.1.617.2 (Delta) and P.1 (Gamma) VOCs. Sera were collected 4 and 16 weeks post-diagnosis. Antibody levels and pseudo-neutralization of the ectodomain of SARS-CoV-2 spike protein/human ACE-2 receptor interaction were analyzed with native, B.1.351, B.1.617.2 and P.1 variant spike proteins. Despite a lower response observed for the variant spike proteins, we report evidence of a sustained humoral response against native, B.1.351, B.1.617.2 and P.1 variant spike proteins among non-hospitalized Canadian adults. Furthermore, this response inhibited the interaction between the spike proteins from the different VOCs and ACE-2 receptor for ≥ 16 weeks post-diagnosis, except for individuals aged 18–49 years who showed no inhibition of the interaction between B.1.617.1 or B.1.617.2 spike and ACE-2. Interestingly, the affinity (K_D_) measured between the spike proteins (native, B.1.351, B.1.617.2 and P.1) and antibodies elicited in sera of infected and vaccinated (BNT162b2 and ChAdOx1 nCoV-19) individuals was invariant. Relative to sera from vaccine-naïve (and previously infected) individuals, sera from vaccinated individuals had higher antibody levels (as measured with label-free SPR) and more efficiently inhibited the spike–ACE-2 interactions, even among individuals aged 18–49 years, showing the effectiveness of vaccination.

## Introduction

The severe acute respiratory syndrome coronavirus 2 (SARS-CoV-2) has infected and caused the death of millions of individuals across the globe since 2019^1^. This RNA coronavirus of zoonotic origin has a diameter of 80–90 nm with several structural proteins, including nucleocapsid and spike^2^. The virus invades and replicates in the lower respiratory tract and causes pneumonia in some infected individuals, which is one of the most frequent complications of the coronavirus disease COVID-19.

The immune system fights the infection by eliciting an innate immune response^3^, and a B- and T-cell mediated response^4,5^. The humoral response against SARS-CoV-2 follows a classical pattern in which IgMs and IgAs are expressed 1–2 weeks post-diagnosis during the recovery phase and IgGs are expressed 2–4 weeks post-diagnosis during the convalescence phase^6,7^. IgGs may be associated with long-term humoral memory as they are detectable several months post-diagnosis^8^. However, antibody levels appear to be lower for asymptomatic or paucisymptomatic individuals compared to those with severe illness requiring hospitalization^9–11^. Given this notable difference, studies are needed to better understand the humoral response of non-hospitalized individuals, a population that remains understudied.

As an abundant surface protein with a large, accessible ectodomain, spike protein is the immunogen of SARS-CoV-2 that elicits the strongest humoral response. As a result, the ectodomain of spike protein (simply referred to as ‘spike protein’ here) forms the basis of current mRNA and viral-vector-based vaccines^12,13^. Spike is a trimeric glycoprotein with each monomer composed of an S1 and S2 subunit. During viral fusion with human cells, the receptor binding domain (RBD) of the S1 subunit binds to the membrane-bound angiotensin-converting enzyme 2 (ACE-2) receptor^14^, and the S2 subunit mediates membrane fusion. ACE-2 is particularly abundant on the surface of lower respiratory tract cells, which makes them susceptible to infection and can cause pneumonia^15,16^. The antibodies produced in response to current vaccines work primarily by binding to the RBD of spike protein, thus blocking its interaction with ACE-2^17^, which is thought to mediate their effectiveness.

Over the course of the pandemic, several SARS-CoV-2 variants of concern (VOCs) have emerged in the United Kingdom (B.1.1.7, also named Alpha variant by the WHO), in South Africa (B.1.351, Beta), in Brazil (P.1, Gamma), and in India (B.1.617.1, Kappa and B.1.617.2, Delta). These VOCs are now the dominant strains worldwide and harbor multiple mutations in the spike protein^18–20^, which raises questions about the effectiveness of the humoral immunity of individuals who were previously infected with the native strain that originated from Wuhan, China (*i.e.,* variant-naïve individuals), and those who were immunized by the first-generation vaccines that use the native spike protein as immunogen. These mutations may affect the ability of antibodies to bind to the virus which may thus evade neutralizing antibodies^21–23^. It has been reported that the N501Y and K417N mutations present in the B.1.1.7, B.1.351 and P.1 VOC spike protein reduce the activity of antibodies from convalescent and post-vaccination serum or therapeutic monoclonal antibodies^24–30^. It is suspected that the multiple mutations harbored by spike protein of the VOCs could lead to a conformational change in the RBD of spike and affect binding to ACE-2^18^. Detailed investigations of the cross-reactivity of antibodies are therefore necessary to evaluate the susceptibility of individuals to infection by the VOCs^31,32^, particularly those infected early during the pandemic as well as those immunized, all of whom are thus presumably naïve to these variants.

Several methodological approaches are available to study the humoral response against SARS-CoV-2. Most serological assays use ELISA to assess the seroprevalence of SARS-CoV-2. These assays typically detect IgGs targeting the S1 subunit or the trimeric spike protein, which improves performance compared with assays that target the RBD^33^. However, detecting anti-spike IgGs alone provides an incomplete picture of the humoral response, as effective antibodies should inhibit the interaction of spike protein with ACE-2^34,35^. Cell-based neutralization assays are the gold standard but require live viruses and thus a biosafety level 3 (BSL3) lab, which makes these assays costly, complex and lengthy to perform. Cell- and virus-free surrogate or pseudo-neutralization assays could provide valuable functional information on the inhibition of the interaction between spike protein and ACE-2, as recently demonstrated by a surrogate ELISA neutralization assay^36^. Sensing techniques such as surface plasmon resonance (SPR) provide complementary biochemical data to ELISA. SPR has been used to conduct serological tests^37^ and to measure various biochemical parameters influencing the strength of the humoral response, including the binding constant of the antibodies to spike or its subunits^38^, the inhibition of the spike protein:ACE-2 interaction by neutralizing antibodies^39,40^ and the equilibrium dissociation constant (K_D_) of recombinant human ACE-2 with the RBD of spike protein^41^.

In this longitudinal study, we assessed the cross-reactivity of antibodies produced by non-hospitalized, variant-naïve SARS-CoV-2-positive individuals against the native spike protein, as well as the B.1.351, B.1.617.2 and P.1 VOC spike proteins. Notably, an in vitro SPR pseudo-neutralization assay was developed to determine the ability of convalescent sera to inhibit the interaction between native or variant spike proteins and ACE-2, including a limited number of individuals in our cohort who were vaccinated in the late stages of the study.

## Results

### Cross-reactivity of antibodies with the native and B.1.351 spike proteins

Serum samples from 32 non-hospitalized individuals who tested positive for SARS-CoV-2 (PCR confirmed on average 17.25 days prior to enrollment) were collected at weeks 4 and 16 post-diagnosis. Inclusion criteria included quotas based on age, so that the cohort consisted of four age groups (18–49, 50–59, 60–69 and 70 + years; n = 8 each) that each comprised eight individuals. A second PCR test was conducted at the time of enrollment, and 7 (22%) individuals had a negative test result, indicating that some had fully recovered while most had not. The results of this second PCR-based diagnostic test did not influence eligibility to the current study. Control sera were collected from eight individuals never diagnosed with SARS-CoV-2. Of note, one individual aged 60–69 years did not report on week 16 (n = 7 for this age group).

**Figure 1:**
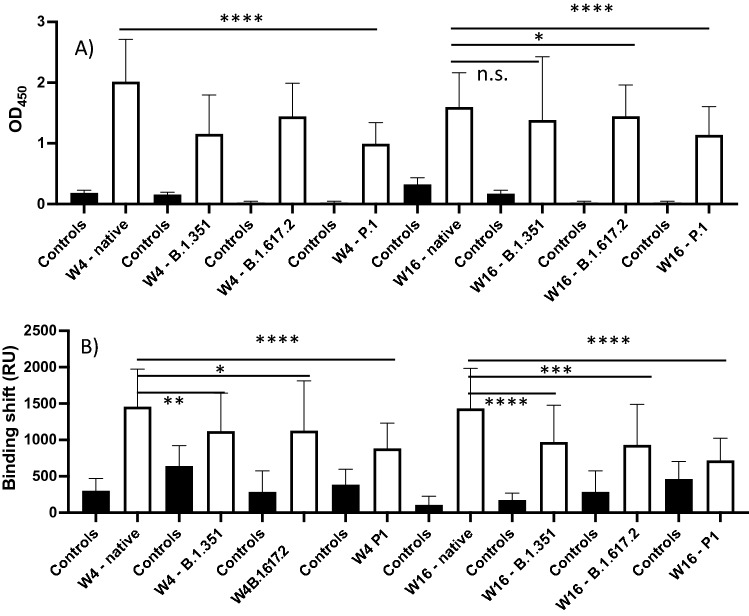
Average ELISA OD_450_ (**A**) and SPR binding shifts (**B**) for the detection of anti-spike IgG in SARS-CoV-2-positive sera (n = 32) at week 4 (W4) and week 16 (W16) post-diagnosis for the native, B.1.351, B.1.617.2, and P.1 spike proteins. Controls are sera from SARS-CoV-2-negative individuals who were never diagnosed with SARS-CoV-2 (n = 8). Error bars represent one standard deviation. n.s., not statistically significant, *p < 0.05, **p < 0.01, ***p < 0.001, and ****p < 0.0001.

ELISA performed well to identify SARS-CoV-2-positive individuals from the negative control group based on the native spike protein (Area under the curve (AUC) = 1.00 and *p* < 0.0001), correctly identifying all positive and negative samples (sensitivity = 100%, specificity = 100%, Tables 1, S1, S2, Figs. 1, S1). ELISA results showed no difference among individuals with a positive test result, whether their second PCR test result was negative or positive at the time of enrollment (14–21 days post diagnosis, Fig. S2). The SPR assays also performed well for the native spike protein (AUC = 0.99 and *p* < 0.0001), correctly identifying all samples with the exception of one positive sample which tested negative at week 4 (sensitivity = 97%, specificity = 100%, Tables 2, S3, S4). Whereas the OD_450_ decreased by approximately 20% from weeks 4 to 16 for the native spike protein, the SPR binding shift did not change significantly (Fig. 1). The ELISA OD_450_ and the SPR shift tended to decrease for B.1.351, B.1.617.2 and P.1 at weeks 4 and 16 in comparison to the native spike protein, the greatest difference being week 4 (Figs. 1, S1). As a result, a few positive samples tested negative when SPR assays were conducted with the B.1.351 spike variant, a tendency that increased with the B.1.617.2 and P.1 spike variants (Table 2). This affected the AUCs (range: 0.75–0.99) and the ability of SPR to differentiate positive samples from the negative controls (Fig. S3). However, the performance of ELISA remained excellent (AUC: 1.00 and *p* < 0.0001 for all VOCs, except for B.1351 at week 16, AUC: 0.99 and *p* < 0.01, Table 1, Fig. S4).

**Table 1:** ELISA for the detection of IgG targeting the native, B.1.351, B.1.617.2, and P.1 spike proteins using serum from variant-naïve, SARS-CoV-2-positive individuals at weeks 4 and 16 post-diagnosis.

		Native spike		B.1.351 spike		B.1.617.2 spike		P.1 spike	
		W4	W16	W4	W16	W4	W16	W4	W16
OD_450_ (A.U)	Pos	2.0 ± 0.7	1.6 ± 0.6	1.4 ± 0.7	1.5 ± 0.8	1.4 ± 0.5	1.4 ± 0.5	1.0 ± 0.3	1.1 ± 0.5
OD_450_ (A.U)	Neg	0.18 ± 0.04	0.32 ± 0.11	0.16 ± 0.04	0.17 ± 0.05	0.02 ± 0.03	0.02 ± 0.03	0.02 ± 0.03	0.02 ± 0.03
Threshold (A.U)		0.4	0.6	0.25	0.2	0.1	0.1	0.1	0.1
COVID + sera	# Pos	32	31	32	31	30	30	31	31
	# Neg	0	0	0	1	0	0	0	0
Control sera	# Pos	0	0	0	1	0	0	0	0
	# Neg	8	8	8	8	8	8	8	8
AUC		1.00	1.00	1.00	0.99	1.00	1.00	1.00	1.00
*p* value		< 0.0001	< 0.0001	< 0.0001	< 0.01	< 0.0001	< 0.0001	< 0.0001	< 0.0001
Sensitivity		100	100	100	96	100	100	100	100
Specificity		100	100	100	88	100	100	100	100

**Table 2:** SPR assay for the detection of human IgG targeting the native, B.1.351, B.1.617.2, and P.1 spike proteins using serum from variant-naïve, SARS-CoV-2-positive individuals at weeks 4 and 16 post-diagnosis.

		Native spike		B.1.351 spike		B.1.617.2 spike		P.1 spike	
		W4	W16	W4	W16	W4	W16	W4	W16
Shift (kRU)	Pos	1.5 ± 0.5	1.4 ± 0.6	1.1 ± 0.5	1.0 ± 0.5	1.1 ± 0.7	0.9 ± 0.5	0.8 ± 0.3	0.7 ± 0.3
Shift (kRU)	Neg	0.3 ± 0.2	0.1 ± 0.1	0.6 ± 0.3	0.2 ± 0.1	0.3 ± 0.3	0.3 ± 0.3	0.4 ± 0.2	0.5 ± 0.2
Threshold (kRU)		0.6	0.35	0.7	0.35	0.7	0.7	0.6	0.7
Positive sera	# Pos	31	31	26	27	22	17	27	16
	# Neg	1	0	6	4	9	14	5	15
Control sera	# Pos	0	0	1	0	1	1	1	1
	# Neg	8	8	7	8	7	7	6	6
AUC		0.99	0.99	0.82	0.93	0.87	0.83	0.90	0.75
*P* value		< 0.0001	< 0.0001	< 0.05	< 0.0001	< 0.01	< 0.01	< 0.001	< 0.05
Sensitivity		97	100	81	87	71	55	84	51
Specificity		100	100	88	100	88	88	86	86

ELISA and SPR experiments were then repeated with the RBD of native and B.1.351 spike. However, the ability of the RBD assays to discriminate between positive to negative samples was generally lower than that obtained using full-length spike protein (Figs. S5–S7 and Tables S5–S8, compared to Tables 1, 2), in agreement with previous reports in which using the S1 subunit or trimeric spike ectodomain protein improved assay performance^2,33,42^. Therefore, the full-length spike protein was used for the remaining experiments. Taken together, these results suggest that the sera of variant-naïve, SARS-CoV-2-positive individuals contain antibodies that cross-react with the B.1.351, B.1.617.2 and P.1 spike variants during at least 16 weeks post-diagnosis, although antibody binding appeared reduced for the variants compared with the native protein (Fig. 1), in agreement with a previous report^43^.

**Figure 2:**
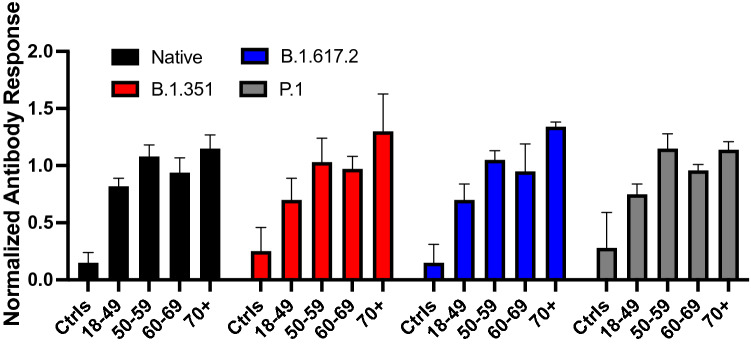
Average normalized antibody binding stratified by age group for human anti-IgGs targeting the native (black), B.1.351 (red), B.1.617.2 (blue) and P.1 (gray) spike proteins among variant-naïve, non-hospitalized individuals. SPR and ELISA data from weeks 4 and 16 post-diagnosis were normalized to the mean of the overall cohort and pooled. A normalized antibody binding of 1 thus refers to a response equivalent to the average of all SARS-CoV-2-positive individuals (n = 8 for each data set) and the error bars correspond to one standard deviation). Ctrls: SARS-CoV-2-negative sera (n = 8).

When stratified by age, antibody binding increased with age for native spike. This trend was even more evident for data acquired with the VOCs spike proteins (Fig. 2). On average, individuals in the 70 + years group exhibited ELISA and SPR responses 15% (native) to 30% (VOCs) above the mean of the overall cohort, those aged 50–59 or 60–69 years exhibited responses within 10% of the mean, and those aged 18–49 years exhibited responses 18% (native) to 30% (VOCs) below the mean.

### Label-free response and affinity of antibodies produced by variant-naïve individuals against native and variant spike proteins

SPR sensing is ideally suited for the measurement of antibodies and proteins in moderately diluted (i.e., 1:10) to undiluted serum^44^. Because SPR is a label-free method, it can be used to study biochemical properties of protein–protein interactions, such as the K_D_. Prior studies have successfully used SPR to study the affinity of antibodies produced following SARS-CoV-2 vaccination^45^ or COVID-19 infection^38^. Hence, we adapted the SPR assay to measure antibody binding and affinity for the native spike protein based on the sera of four SARS-CoV-2-positive individuals in each of the four age groups; we also determined affinity for the B.1.351 variant spike protein for the individuals aged 18–49 years (Fig. S8). As a result of vaccination roll-out in late 2020/early 2021, a limited number of the SARS-CoV-2-positive individuals in our cohort were vaccinated in the late stages of the study (Table S12). Their sera samples were acquired 24 weeks post-diagnosis and at least 2 weeks post-vaccination. Analysis of five of these allowed for a first glance into the impact of vaccination on affinity of their antibody development.

**Figure 3:**
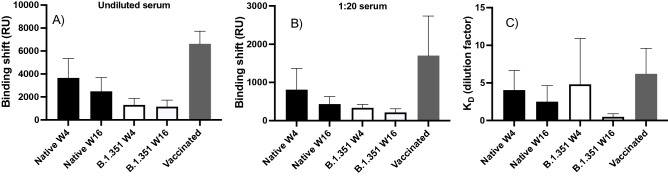
SPR binding shift for undiluted serum (**A**) and serum at a 1:20 dilution (**B**) from SARS-CoV-2-positive individuals at weeks 4 (W4) and 16 (W16) post-diagnosis for the native (black, n = 16, 4 age groups) and B.1.351 (white, n = 4, 18–49 years) spike proteins, and for vaccinated individuals (gray, n = 5) for the native spike protein. (**C**) A dissociation constant was estimated from the dilution series assuming a 1:1 binding model according to the data presented in panels (**A**) and (**B**). The dilution series used in SPR data acquisition consisted of the following: 1:40, 1:20, 1:10, 1:5, 1:2.5 and undiluted serum. In all panels, error bars represent one standard deviation.

The SPR binding shift for the undiluted sera decreased slightly between weeks 4 and 16 for the native spike protein (*p* = 0.03) and remained constant for B.1.351 (*p* = 0.73) spike protein (Fig. 3). The SPR binding shift decreased from weeks 4 to 16 when using a 1:20 dilution for the native spike protein (*p* = 0.02) and tended to decrease for the B.1.351 (*p* = 0.11) spike protein. As shown earlier, a similar decrease was obtained using ELISA (Fig. 1), which was performed using samples at a greater dilution (1:50). For the B.1.351 variant, the SPR signal appeared modestly lower (although not statistically significant at week 4) than that observed for native spike at week 4 (*p* = 0.11) and week 16 (*p* = 0.05; Fig. 3). In comparison, vaccinated individuals exhibited a twofold higher SPR response for both 1:20 dilution (*p* = 0.02) and undiluted samples (*p* < 0.01).

The label-free SPR assay revealed that binding shift increased almost linearly with serum dilution (Fig. S8—left panel), and K_D_s were extracted by fitting a 1:1 binding site Langmuir isotherm (Fig. S8—right panel). Because the serum concentration is expressed as a dilution factor (i.e., a high dilution factor corresponds to low antibody levels), a high K_D_ corresponds to a high affinity of the antibodies for spike. Attempts to extract the association (k_on_) and dissociation (k_off_) rates were unsuccessful since sera contain a mixture of polyclonal antibodies. The estimated K_D_ appeared to decrease with time for the two spike proteins, but the difference was not statistically significant (native: *p* = 0.08 and B.1.351: *p* = 0.29) between weeks 4 to 16 (Fig. 3C). No significant difference in K_D_ was observed between native and B.1.351 spike proteins at week 4 (*p* = 0.71), week 16 (*p* = 0.13) or with vaccinated individuals (*p* = 0.15).

### Pseudo-neutralization SPR inhibition assay

**Figure 4:**
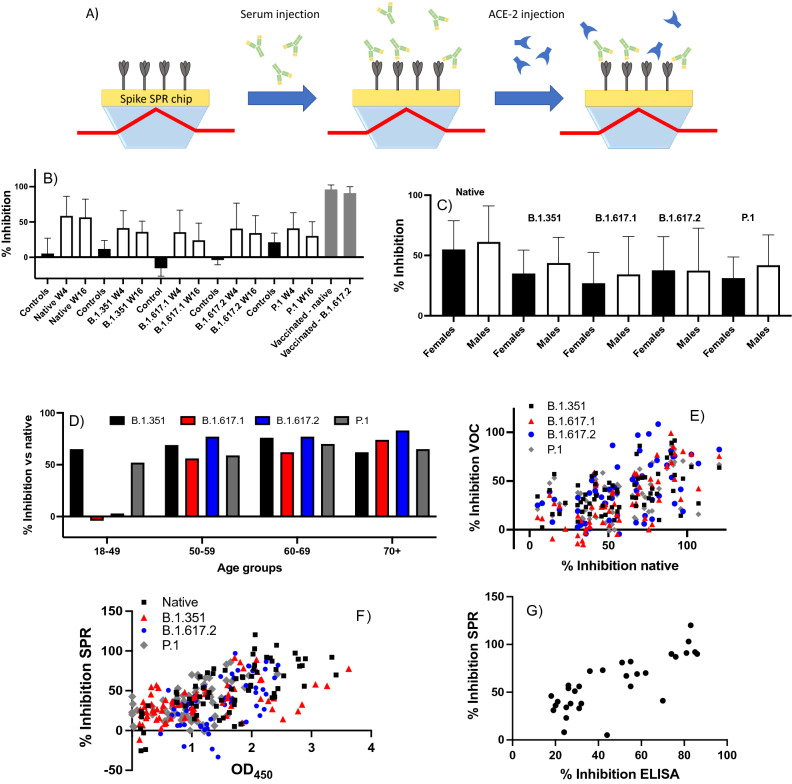
(**A**) Schematic illustrating the principle of the SPR pseudo-neutralization assay to quantify the inhibition of the interaction between spike protein and ACE-2 in the presence of SARS-CoV-2-positive sera. Serum from an individual who tested positive for SARS-CoV-2 is injected onto an SPR chip that has been functionalized with spike protein, and a preparation of recombinant human ACE-2 is subsequently injected. A reduced SPR response (and thus higher percent inhibition) is observed if the serum antibodies inhibit the spike–ACE-2 interaction. (**B**) Observed inhibition of the interaction of ACE-2 with native, B.1.351, B.1.617.2, and P.1 spike proteins by variant-naïve SARS-CoV-2-positive sera (n = 32). Controls: SARS-CoV-2-negative sera (n = 8). Error bars represent one standard deviation. (**C**) Observed inhibition of the interaction of ACE-2 with native, B.1.351, B.1.617.2, and P.1 spike proteins by variant-naïve SARS-CoV-2-positive sera as a function of sex (females: n = 18; males: n = 14). (**D**) Normalized inhibition for the B.1.351, B.1.617.1, B.1.617.2 and P.1 variants expressed as a percentage of inhibition compared to the native spike protein for the same age group (n = 8 for each age group). Data from weeks 4 and 16 are pooled. (**E**) Correlation between the percent inhibition (for weeks 4 and 16 post-diagnosis combined) observed for native and for the B.1.351, B.1.617.1, B.1.617.2 and P.1 spike proteins; each dot corresponds to an average of 2 replicates for a single individual (n = 31 or 32 depending on the data sets). (**F**) Correlation between the ELISA OD_450_ results (Fig. 1 and Tables S1, S2) and pseudo-neutralization results for weeks 4 and 16 combined (n = 31 or 32 depending on the data sets). (**G**) Correlation between the ELISA and SPR pseudo-neutralization results obtained with the native spike protein for the samples at week 4 (n = 31).

To better understand the potential functional implications of antibody cross-reactivity, we adapted a previously reported in vitro pseudo-neutralization assay^39,46^ to measure how antibody binding to spike protein affects its interaction with ACE-2 (details in supporting information, Schemes S1 and S2, Figs. S9–S11 and Tables S9, S10). The difference in inhibition of the spike–ACE-2 interaction between weeks 4 and 16 post-diagnosis was not statistically significant for the native (mean inhibition %: week 4 = 59%, week 16 = 57%, *p* = 0.67), B.1.351 (week 4 = 41%, week 16 = 36%, *p* = 0.1), and B.1.617.2 spike proteins (week 4 = 41%, week 16 = 34%, *p* = 0.1), while statistically significant reductions were observed for the B.1.617.1 (week 4 = 36%, week 16 = 24%, *p* < 0.01) and P.1 spike proteins (week 4 = 41%, week 16 = 30%, *p* < 0.01; Table S11). Sera from variant-naïve SARS-CoV-2-positive individuals inhibited the spike–ACE-2 interaction significantly less for all variants compared to the native spike protein at week 4 (B.1.351, *p* < 0.0001; B.1.617.1, *p* < 0.0001; B.1.617.2, *p* < 0.01; P.1, *p* < 0.0001; Fig. 4 and Table S11) and at week 16 (B.1.351, *p* < 0.001; B.1.617.1, *p* < 0.0001; B.1.617.2, *p* < 0.001; P.1, *p* < 0.0001; Fig. 4 and Table S11). No significant difference in inhibition was observed between female and male individuals for the native (females = 55%, males = 61%, *p* = 0.37), B.1.351 (females = 35%, males = 44%, *p* = 0.10), B.1.617.1 (females = 27%, males = 34%, *p* = 0.31), B.1.617.2 (females = 38%, males = 37%, *p* = 0.98), and P.1 (females = 31%, males = 42%, *p* = 0.051) spike proteins (Fig. 4C). The normalized inhibition for the variants relative to the native spike protein was relatively constant (range: 56–83%, Fig. 4D) for the different age groups, except for the 18–49 age group which showed no inhibition (normalized inhibition of − 4% and 3%) for the two variants in the B.1.617 lineage. The percent inhibition observed for each individual showed only a mild correlation between native and B.1.351 spike proteins (r = 0.46; Fig. 4E). However, the percent inhibition correlated well with the ELISA OD_450_ results of the native spike proteins (r = 0.70) and moderately well with the VOCs (B.1.351: r = 0.52, B.1.351: r = 0.54, and P.1: r = 0.51), suggesting that anti-spike IgG concentration is a predictor of the inter-individual variation in the ability of sera to inhibit the interaction between spike protein and ACE-2 (Fig. 4F). The percent inhibition obtained with SPR also correlated well (r = 0.78) with the results of a similar pseudo-neutralization assay performed with ELISA (Fig. 4G), adapted from a recently reported protocol^47^. Finally, the cohort of nine vaccinated individuals (BNT162b2 n = 8 and ChAdOx1 nCoV-19 n = 1) showed significantly higher percent inhibition to the native (96% ± 6%, *p* < 0.001 vs infected only) and B.1.617.2 (91% ± 9%, *p* < 0.001 vs infected only) spike proteins in comparison to vaccine-naïve infected individuals (Fig. 4B, Table S12).

## Discussion

The protein–protein interaction data reported in this study show cross-reactivity of antibodies produced by variant-naïve, PCR-confirmed SARS-CoV-2-positive individuals against the B.1.351, B.1.617.2, and P.1 VOCs. Notably, none of the SARS-CoV-2-positive individuals included in this study experienced severe symptoms that required hospitalization; this population remains understudied in the existing literature.

Age correlated well with antibody binding: individuals aged 70 + years generally had higher than average ELISA OD_450_ and SPR responses, those younger than 49 years exhibited below average responses, and those aged 50–69 years exhibited intermediate responses (Fig. 2). This was exacerbated with the Delta variant (B.1.617.2) and its closely-related B.1.617.1 variant. Convalescent sera from individuals younger than 49 years were not able to inhibit the interaction of the spike protein of those two variants with ACE-2, a result in agreement with a recent study showing that the B.1.617.2 variant was less sensitive to sera from naturally immunized individuals^20^. Other studies also found that age is positively correlated with antibody levels as measured by ELISA among individuals hospitalized for COVID-19^48^ or any cause (whether it be COVID-19 or any other condition)^49^. Our study builds on this evidence by showing a similar correlation for the spike proteins of the B.1.351, B.1.617.2 and P.1 VOCs.

The higher ELISA and SPR signals observed against native spike protein than spike proteins for VOCs (Fig. 1, Tables 1, 2) are consistent with the period of our sample acquisition, prior to reports of any VOC and were thus presumably infected with the native strain. This agrees with a report suggesting that antibodies produced by variant-naïve individuals cross-reacted with the B.1.351 spike protein, albeit at lower titer^50^.

Furthermore, K_D_s (expressed in units of dilution) appeared to be higher (therefore tighter binding) at week 4 than week 16 for native and B.1.351 spike proteins, but the difference was not statistically different. Further data would need to be acquired to validate if there is presence of greater affinity of antibodies to all the spike proteins tested earlier post-diagnosis. Taken together, these results suggest that the affinity of antibodies wanes between weeks 4 and 16 post-diagnosis, and that antibody binding levels were lower in sera of individuals 16 weeks post-diagnosis. Our confirmation that the antibodies elicited in variant-naïve, SARS-CoV-2-positive individuals effectively cross-react with B.1.351, B.1.617.2 and P.1 spike protein up until at least 16 weeks post-diagnosis agrees with other reports^8,51–55^.

While informative, biochemical data on antibody binding provide only a partial picture of the humoral response. The pseudo-neutralization SPR assay showed that convalescent sera effectively inhibited the interaction between ACE-2 and the native or B.1.351, B.1.617.1, B.1.617.2 and P.1 spike proteins. The decrease in inhibition for the VOCs in comparison to the native spike protein agrees with a recent report on vaccinated individuals which used a lentiviral spike pseudotyped virus assay^56^. Furthermore, the degree of inhibition correlated with the observed ELISA response for anti-spike protein IgGs (Fig. 4), suggesting that antibody levels explain a large proportion of the inter-individual variation in the ability of sera to inhibit the spike–ACE-2 interaction. Consistent with the ELISA response being higher for the native protein, inhibition of this interaction was more important for the native spike protein. Other reports found that the levels of neutralizing antibodies decreased within 3 months post-diagnosis^52,57–59^, which was not seen here in non-hospitalized individuals. One of those reports associated IgMs with improved neutralization early post-diagnosis^57^. While such association with antibody isotype cannot be established with our data, the antibody levels and the apparent decrease in K_D_ from weeks 4 to 16 post-diagnosis tend to support that antibody affinity is more efficient shortly after infection. These results suggest that antibodies from convalescent sera can inhibit the interaction of spike protein with ACE-2, although the degree of inhibition was larger for native spike protein given that included individuals were presumably infected with the native strain of SARS-CoV-2.

Label-free SPR measurements indicated that vaccination led to an increase in antibody levels in previously infected individuals. However, higher antibody levels did not appear to improve the K_D_ of the antibodies in vaccinated individuals. Vaccination improved inhibition of the spike–ACE-2 interaction, seeing > 90% percent inhibition in sera of vaccinated individuals compared to < 60% for vaccine-naïve individuals. Interestingly, one previously infected individual received two doses of vaccine over the 24-week follow-up period (i.e. at weeks 5 and 20), allowing for the assessment of the humoral response before and after vaccination. Of note, this individual was aged 18–49 years, an age group that showed a relatively poor inhibition of the spike–ACE-2 interaction (especially that involving the B.1.617.2 spike protein). At weeks 2 and 4 (i.e. before vaccination, post-infection), the percent inhibition of this individual was < 20% for the native and B.1.617.2 spike proteins (Fig. S12), in agreement with the low percent inhibition observed in that age group (Fig. 4D, Table S11). After week 4 (i.e. post-vaccination), the percent inhibition increased > 80% and was sustained for at least 20 weeks following vaccination for both the native and B.1.617.2 spike proteins (Fig. S12). These data provide further evidence that vaccination is beneficial to elicit neutralizing antibodies to the B.1.617.2 variant, which is currently dominant across the world.

This study has some limitations, including the limited number of enrollees and the small number of time points assessed post-diagnosis. Increasing sample size should improve the representativeness for some of the experiments. Furthermore, it remains unclear whether ELISA and SPR data may be used to predict the effectiveness of an individual’s humoral response to prevent a new infection, whether from the native strain or from a variant strain, since clinical outcomes following a subsequent SARS-CoV-2 exposure were not collected. As vaccination progresses, this question will also apply to immunized individuals. Despite the in vitro nature of these experiments, cell-based assays should yield largely similar results since the results of another in vitro, surrogate neutralization assay were found to strongly correlate with those of a cell-based neutralization assay^36,60,61^.

In conclusion, antibodies in sera of SARS-CoV-2-positive, variant-naïve individuals cross-reacted with the spike protein of the B.1.351, B.1.617.2 and P.1 VOCs, albeit with a decrease in antibody binding as measured by ELISA and SPR. Antibody levels decreased from weeks 4 to 16 and were positively correlated with age. SPR results suggested that the higher affinity and higher concentration antibodies wane from weeks 4 to 16 post-diagnosis. However, remaining antibodies effectively inhibited the interaction between native and variant spike proteins and recombinant human ACE-2. ELISA results correlated with the degree of inhibition, suggesting that high antibody levels are needed for optimal pseudo-neutralization. Vaccination increased the antibody levels and strongly improved the percent inhibition of the spike–ACE-2 interaction. Taken together, these results suggest that variant-naïve, non-hospitalized, SARS-CoV-2-positive individuals have sustained humoral immunity at later times post-diagnosis and that vaccination improves the humoral response to SARS-CoV-2.

## Experimental section

### Materials

N-Ethyl-N′-(3-dimethylaminopropyl)carbodiimide hydrochloride, (EDC, crystalline, cat. no. E6383), N-hydroxysuccinimide (NHS, 98%, cat. no. 130672), ethanolamine hydrochloride (≥ 99.0%, cat. no. E6133), glycine hydrochloride (≥ 99.0%, cat. no. G2879), bovine serum albumin (≥ 98.0%, cat. no. 5470), Tween20 (cat. no. P.1379), and human AB serum (cat. no. H4522) were obtained from Sigma Aldrich. The running buffer was composed of phosphate buffer saline 1X (VWR, cat. no. L0119), 0.1% BSA, and 0.005% Tween20. Goat anti-human IgG (Jackson Immunoresearch, cat. no. 109-005-003) and human recombinant ACE-2 (Sino Biologicals, cat. no. 10108-H08H) were obtained from commercial sources. Native (PRO1-429 (SmT1-1^62^), B.1.351 (PRO6429-1 (SmT1 (SA))), B.1.617.1 (PRO7109-1 (SmT1 (B.1.617.1))), B.1.617.2 (PRO7176-1 (SmT1 (B.1.617.2))) and P.1 (PRO6875-2 (SmT1v3 (BR))) spike proteins and biotin-ACE-2 (PRO5436-5 (SH6F-ACE2-BAP)) were obtained from the National Research Council of Canada and expressed based on the protocols reported elsewhere^63^.

#### ELISA assays

Semi-quantitative ELISA was performed based on the protocols of Krammer and of Finzi and Bazin^11,64,65^, as recently reported^37^. The sera were heat inactivated for 60 min at 55 °C in a heating block and diluted 1:50 before use. ELISA operates at a greater dilution factor (1:50) compared to SPR (1:5). Therefore, ELISA preferentially measures high concentration and high affinity (high K_D_) antibodies, whereas SPR measures both high and low concentration or high and low affinity antibodies.

The relevant SARS-CoV-2 antigenic spike protein was diluted in PBS at a concentration of 2.5 μg/mL. Immulon 1B 96-well plates (Thermo Fischer Scientific) were coated with 100 μL of diluted antigen and incubated at 4 °C overnight. In parallel, clinical samples were inactivated for 1 h at 56 °C in a heating block, then kept at 4 °C overnight. The following day, plates were washed 4 times with PBS-T using a 50 TS Microplate Washer (Biotek) automated plate washer followed by addition of 300 μL of blocking solution (PBS-T + 3% (w/v) milk powder) to each well. After 1 h of incubation at RT, plates were washed 4 times with PBS-T. Serum samples were diluted 1:50 in PBS with 0.1% Tween20 and 1% milk powder, in a 96-well polystyrene dilution plate and 100 μL was added to each well. Plates were incubated for 1 h at RT and washed 4 times with PBS-T. The secondary antibody (100 μL of 1:10,000 dilution Anti-Human IgG (gamma-chain specific)-Peroxidase antibody produced in goat, Sigma-Aldrich, cat. no. A6029-1ML) was added. Plates were incubated for 1 h at RT then washed 4´ with PBS-T. Addition of 100 μL of TMB (3,3',5,5'-tetramethylbenzidine, Sigma-Aldrich) to each well was followed by a 20 min incubation at RT. Color development was initiated by addition of 100 μL of 2 M HCl. Absorbance was immediately recorded at 450 nm and 595 nm (plate background, subtracted from absorbance at 450 nm) in a FLUOstar Optima microplate reader (BMG Labtech).

### SPR measurements for IgG detection

The methods for the SPR detection of human IgG antibodies were described recently^37^ using a portable SPR instrument^66^ (Affinité Instruments, Canada). In brief, after NHS and EDC activation for 2 min, the spike proteins or RBD for the native strain of SARS-CoV-2 or the B.1.351 variants were immobilized at a concentration of 20 µg/mL for 20 min in pH 4.5 acetate buffer and the sensors were washed with 1 M ethanolamine pH 8.5 (10 min) and running buffer composed of pH 7.4 PBS (137 mM NaCl, 10 mM phosphate, 2.7 mM KCl, pH 7.4) supplemented with 0.1% bovine serum albumin (BSA) and 0.005% Tween 20 as described previously. Serum samples were diluted 1:5 in the running buffer and injected for 10 min. Following a quick wash with running buffer, secondary detection was performed for 10 min with a 40 µg/mL solution for the spike protein and 20 µg/mL for RBD of AffiniPure goat anti-human IgG (H + L). The surface was regenerated with 10 mM glycine pH 2.2 solution for a few seconds and washed with running buffer before the next set of sera were injected. Experiments were performed with the SPR instrument inside a laminar flow cabinet in a biosafety Level 2 (BSL2) laboratory. The SPR instrument had 4 independent channels on the fluidic cell allowing the measurement of up to 4 samples in a single run.

### Antibody binding affinity

For affinity measurements using SPR, sera were diluted in the running buffer with dilutions ranging from 1:40 to no dilution (undiluted serum). The sera were then injected sequentially from the greatest dilution to the undiluted sera. Samples were measured in triplicate and the reference channel was used for the background response correction due to nonspecific binding of negative sera at the same dilution factors. In this case, the fluidic cell of the SPR system was identical to that previously described by Zhao et al.^66^. Data collected at equilibrium were fit to a single binding site Langmuir model to extract a K_D_ in dilution titer.

### Surrogate inhibition assay

A SPR assay was designed to serve as an in vitro surrogate to cell-based neutralization assay. The spike proteins for the native strain of SARS-CoV-2 or the B.1.351 variant were immobilized on the SPR chip as described above. Following the passivation of the surface with ethanolamine, the SPR sensor was equilibrated in a commercial serum exempt of anti-spike antibody diluted 1:5 in running buffer for typically 10–15 min until a stable baseline. Then, different samples were injected on the four channels of the SPR instrument. On two channels, sera from an individual in the negative control group or sera from a PCR-confirmed SARS-CoV-2 infected individual (4- and 16-weeks post infection were tested) diluted 1:5 in running buffer were reacted with the spike protein for 10 min. Following a quick wash with running buffer for a few seconds, human recombinant ACE-2 was then injected at a concentration of 5 µg/mL for 10 min. If antibodies blocking the interaction of human recombinant ACE-2 with the spike protein were present, a lower SPR response was recorded; signals were compared to a positive and a negative control run in the other two channels. The positive control consisted of an identical SPR chip but using a SARS-COV-2-negative, pooled commercial serum and for which human recombinant ACE-2 was then injected to obtain the maximum SPR signal from the human recombinant ACE-2–spike interaction. The negative control consisted in the injection of the same serum sample diluted 1:5 with running buffer as in the measurement channels, but with the injection of running buffer (no human recombinant ACE-2) in otherwise identical conditions; the background response was subtracted from the measurements. No heat treatment was applied to the sera to ensure native conditions for the measurement of pseudo-neutralization.

### Clinical samples

Adult volunteers were recruited after written informed consent at the Centre Hospitalier Universitaire de Québec—Université Laval (CHUL, approved by the “Comité d’éthique de la recherche du CHU de Québec-UL”, Registration Number 2021-5241) in Quebec City, Canada. All experiments were performed in accordance with relevant guidelines and regulations. Sera from the same cohort of individuals as previously reported were used in this study^37^. Blood samples were collected and processed to obtain the sera as previously described^37^. All nasopharyngeal samples (outpatients and inpatients) were PCR-tested for COVID-19 at a reference laboratory early during the pandemic, but prior to enrollment. The database storing these results was searched, and the patients who had a positive nasopharyngeal PCR test result for COVID-19 (index PCR) were sent a letter explaining the study and giving contact information if they were interested to participate. Interested participants were enrolled if they satisfied the following criteria: aged > 18 years, and neither hospitalized nor admitted to intensive care unit at the time of enrollment. Individuals were recruited in four age group: 18–49, 50–59, 60–69 and 70 + years of age at the time of enrollment; they had received a positive PCR diagnosis (index PCR) for COVID-19 four weeks prior to serum collection and had blood drawn at 16 weeks post infection. Of these, eight samples were randomly selected in each age group for a total of 32 participants, 14 males and 18 females. In one case, a volunteer in the 60–69 age group did not provide a sample on week 16, resulting in *n* = 7 for that data point. One control nasopharyngeal PCR was done 14–21 days post index PCR (average of 17.25 days); 78% of the participants (25/32) still had a positive PCR. Participants were enrolled regardless of the result of this control PCR. One participant had no symptom related to COVID-19. All other volunteers^31^ were considered mildly symptomatic with an average of 3 symptoms among fever, myalgia, headache, sore throat, new olfactory or taste disorder, cough or difficulty breathing. Negative controls were collected from eight individuals (age range: 20–55 and median: 47.5 years of age, 7 females and 1 male) having never received a COVID-positive test. Enrollment was completed prior to October 1st 2020. According to the *Institut National de Santé Publique du Québec* (INSPQ, Quebec National Public Health Institute), the local public health authority where the study was conducted, the first cases of the B.1.351 VOC was reported in early February 2021^67^, while the other variants (B.1.617.1, B.1.617.2 and P.1) were reported even later. Hence, all individuals in the current study were infected with the native SARS-CoV-2 strain originating from Wuhan. In all cases, sera from 8 individuals in the following age groups (18–49, 50–59, 60–69 and 70 + years old) were compared to 8 negative controls from individuals with no confirmed exposure to SARS-CoV-2. In addition to the vaccine-naïve individuals reported above, nine previously infected individuals were vaccinated during the study with either the ChAdOx1 nCoV-19 or the BNT162b2 vaccines and subsequently provided study-related post-vaccination samples. One of these individuals provided six samples between weeks 2 to 24 following a PCR positive SARS-CoV-2 tests, where vaccination occurred on weeks 5 and 20 post diagnosis.

### Statistics

Statistical values (AUC and *p* values) were calculated with GraphPad Prism version 9.1.0. Means were compared with paired or unpaired two-tailed t-tests when appropriate. The thresholds were generally established as the response from the mean of the controls plus two standard deviations.

## Supplementary Information

Supplementary Information.
