# Author Correction: Cross-reactivity of antibodies from non-hospitalized COVID-19 positive individuals against the native, B.1.351, B.1.617.2, and P.1 SARS-CoV-2 spike proteins

**DOI:** 10.1038/s41598-021-02484-9

**Published:** 2021-11-19

**Authors:** Maryam Hojjat Jodaylami, Abdelhadi Djaïleb, Pierre Ricard, Étienne Lavallée, Stella Cellier-Goetghebeur, Megan-Faye Parker, Julien Coutu, Matthew Stuible, Christian Gervais, Yves Durocher, Florence Desautels, Marie-Pierre Cayer, Marie Joëlle de Grandmont, Samuel Rochette, Danny Brouard, Sylvie Trottier, Denis Boudreau, Joelle N. Pelletier, Jean-Francois Masson

**Affiliations:** 1 Department of Chemistry, Québec Centre for Advanced Materials (QCAM), Regroupement Québécois sur les Matériaux de Pointe (RQMP), and Centre Interdisciplinaire de Recherche sur le Cerveau et l’apprentissage (CIRCA), Université de Montréal, CP 6128 Succ. Centre-Ville, Montréal, QC H3C 3J7 Canada; 2 Department of Chemistry, Department of Biochemistry and PROTEO, The Québec Network for Research On Protein Function, Engineering and Applications, Université de Montréal, CP 6128 Succ. Centre-Ville, Montréal, QC H3C 3J7 Canada; 3 Mammalian Cell Expression, Human Health Therapeutics Research Centre, National Research Council Canada, Montréal, QC Canada; 4 Héma‐Québec, Affaires médicales et innovation, 1070, avenue des Sciences‐de‐la‐Vie, Québec, QC G1V 5C3 Canada; 5 Centre de recherche du Centre hospitalier universitaire de Québec and Département de microbiologie-infectiologie et d’immunologie, Université Laval, 2705, boulevard Laurier, Québec, QC G1V 4G2 Canada; 6 Department of Chemistry and Centre for Optics, Photonics and Lasers (COPL), Université Laval, 1045, av. de la Médecine, Québec, QC G1V 0A6 Canada

Correction to: *Scientific Reports* 10.1038/s41598-021-00844-z, published online 08 November 2021

The original version of this Article contained an error in the spelling of the author Stella Cellier-Goetghebeur which was incorrectly given as Stella Cellier-Goethebeur.

The original Article and accompanying Supplementary Information file have been corrected.

