# Supplementary material for: Cross-reactivity of antibodies from non-hospitalized COVID-19 positive individuals against the native, B.1.351, B.1.617.2, and P.1 SARS-CoV-2 spike proteins
Source: Sci Rep. 2021 Nov 8;11:21601. doi: 10.1038/s41598-021-00844-z (PMC8575961; doi:10.1038/s41598-021-00844-z)
Supplement: Supplementary file 1 — Supplementary Information. [file 41598_2021_844_MOESM1_ESM.docx]

**Supporting information for:**

**Cross-reactivity of antibodies from non-hospitalized COVID-19 positive individuals against the native, B.1.351, B.1.617.2, and P.1 SARS-CoV-2 spike proteins**

Maryam Hojjat Jodaylami^[a] ⊥^, Abdelhadi Djaïleb^[b]⊥^, Pierre Ricard^[a]⊥^, Étienne Lavallée^[b]^, Stella Cellier-Goetghebeur^[b]^, Megan-Faye Parker^[b]^, Julien Coutu^[a]^, Matthew Stuible^[c]^, Christian Gervais^[c]^, Yves Durocher^[c]^, Florence Desautels^[d]^, Marie-Pierre Cayer^[d]^, Marie Joëlle de Grandmont^[d]^, Samuel Rochette^[d]^, Danny Brouard^[d]^, Sylvie Trottier^[e]^, Denis Boudreau^[f]^, Joelle N. Pelletier*^[b]^, and Jean-Francois Masson*^[a]^

*[a] Department of Chemistry, Quebec Centre for Advanced Materials (QCAM), Regroupement Québécois sur les Matériaux de Pointe (RQMP), and Centre interdisciplinaire de recherche sur le cerveau et l’apprentissage (CIRCA), Université de Montréal, CP 6128 Succ. Centre-Ville, Montreal, Québec, Canada, H3C 3J7*

*[b] Department of Chemistry, Department of Biochemistry and PROTEO, The Québec Network for Research on Protein Function, Engineering and Applications, Université de Montréal, CP 6128 Succ. Centre-Ville, Montreal, Québec, Canada, H3C 3J7*

*[c] Mammalian Cell Expression, Human Health Therapeutics Research Centre, National Research Council Canada, Montréal, Québec, Canada.*

*[d] Héma‐Québec, Affaires médicales et innovation, 1070, avenue des Sciences‐de‐la‐Vie, Québec City, Québec, Canada, G1V 5C3*

*[e] Centre de recherche du Centre hospitalier universitaire de Québec and Département de microbiologie-infectiologie et d’immunologie, Université Laval 2705, boulevard Laurier, Québec City, Québec, Canada G1V 4G2*

*[f] Department of Chemistry and Centre for Optics, Photonics and Lasers (COPL), Université Laval, 1045, av. de la Médecine, Québec City, Québec, Canada, G1V 0A6*

* Corresponding authors: [joelle.pelletier@umontreal.ca](mailto:joelle.pelletier@umontreal.ca); tel: +1-514-343-2124

[jf.masson@umontreal.ca](mailto:jf.masson@umontreal.ca); tel: +1-514-343-7342

^⊥^ Authors that contributed equally to the work.

**Table of contents:**

1. RBD SPR and ELISA assays
2. Optimization of the ACE-2:spike inhibition assay
3. Supporting Tables
4. Supporting Figures

**1) RBD SPR and ELISA assays**

A decrease in the ELISA OD_450_ (generally > 1 for spike and <0.5 for RBD, Table S3) and in the SPR shifts (generally >1000 RU for spike and < 400 RU for RBD, Table S4) compared to spike proteins led to lower sensitivity (Tables S5 and S6) and specificity (Tables S5 and S6), in addition to poorer AUC for ROC curves (0.57 to 0.99 for ELISA and 0.58 to 0.89 for SPR, Figure S5 and S6). These poorer performance metrics were obtained for the native RBD as well as for the RBD for the VOCs.

**2) Optimization of the ACE-2:spike inhibition assay**

*Human recombinant ACE-2 immobilization*

We initially tried to immobilize human recombinant ACE-2 to the surface of the SPR sensor and expose the surface to a spike protein solution to the chip for binding. In that scheme, spike would be added to the clinical sera to measure the inhibition of the human recombinant ACE-2−spike protein interaction in the presence of neutralizing antibodies (Scheme S1). The immobilization of human recombinant ACE-2 (conditions: 10 µg/mL, buffer: 10 mM acetate pH 4.5) led to shifts on the order of 1000 RU and the detection of the 2.5 µg/mL native spike protein showed shifts on the order of 200-300 RU, revealing that a functional human recombinant ACE-2 SPR chip could be produced. Regeneration of the chip was possible in 0.1% SDS. However, attempts to perform an inhibition assay were suboptimal with 10 µg/mL spike protein, as we did not see strong inhibition (10%) of the ACE-2−spike protein interaction. In this assay, the spike protein was added to a COVID-positive serum diluted 1:5 in the running buffer, such that neutralizing antibodies should prevent the spike protein from binding to the human recombinant ACE-2-functionalized SPR chip. Decreasing the concentration of spike protein to 2.5 µg/mL led to slightly better performance, obtaining 34% inhibition of the response from the COVID-positive serum. The improvement of the inhibition was to be expected as a decreased concentration of spike should lead to larger inhibition, as a larger proportion of the spike protein should be bound to the antibodies. However, the range, or contrast, of the inhibition assay with human recombinant ACE-2 on the SPR chip was insufficient and further decreasing the concentration of spike would lead to weak SPR signals. As such, the design of the assay was modified to immobilize spike on the surface.

**Scheme S1.** Human recombinant ACE-2 was immobilized on the SPR sensor. Then, a concentration of spike protein was added to a 1:5 diluted sera and pre-incubated for 2 hours. This solution was injected on the SPR sensor and free spike protein could bind to recombinant human ACE-2. In the presence of neutralizing antibodies, the concentration of free spike available to bind to recombinant human ACE-2 decreased and led to a lower response of the SPR sensor. The response was then compared to that obtained in a control serum having no anti-spike antibodies.

*Spike immobilization*

Immobilizing spike on the SPR surface led to improved performance of the inhibition assay. In this case, the spike-functionalized SPR surface (immobilized at 20 µg/mL, buffer: 10 mM acetate pH 4.5 buffer) was first exposed to the COVID-positive sera (diluted 1:5) to bind the spike protein with neutralizing antibodies. Then, a 2.5 µg/mL solution of human recombinant ACE-2 was injected to the surface where it bound to the spike proteins that were not bound to neutralizing antibodies. The reference channel was exposed to a commercial serum exempt of anti-spike antibodies at the same dilution factor and then, exposed to the same concentration of human recombinant ACE-2, providing a positive control (Scheme S2). In those conditions, a decrease of 450 RU of the human recombinant ACE-2 / spike interaction was observed for the COVID-positive serum diluted 1:5, a significant improvement from the 100 RU signal observed with ACE-2 immobilized on the surface.

We then sought to find the optimal dilution factor for the interaction of human recombinant ACE-2. As previously observed with the affinity curves and shown here in Figure S10, the SPR response from anti-spike antibodies in serum (response subtracted from a blank serum) led to a relatively linear increase of the SPR response with dilution. Similarly, a smaller serum dilution, implying a larger anti-spike antibody concentration, led to a relatively linear increase of the inhibition of human recombinant ACE-2 binding to the spike-coated SPR chip. Note that the data in Figure S10 – right panel is presented as a decrease in human recombinant ACE-2 binding, resulting from inhibition of binding to spike protein by the antibodies. A 1:5 dilution was selected with consideration of the volume of serum available and to maximize signal from ACE-2 inhibition. To increase the contrast, human recombinant ACE-2 concentration was increased to 5 µg/mL.

*Measurement of human recombinant ACE-2 interaction with spike*

We ensured that the SPR sensor was functional with the estimation of the K_D_ at 38, 33, 31 and 38 nM with the native, B.1.351, P.1 and B.1.617.2 spike proteins respectively (Figure S11 and Table S7). The difference in K_D_ between the results presented here and the ones of ref (*1*) (<10 nM) may be explained by the difference in protein source, protein structure (RBD (*1*) vs trimeric spike here) and orientation of the protein immobilized on the SPR surface. Analysis of the association (k_on_) and dissociation (k_off_) rates revealed that they were slightly slower for B.1.351, P.1 and B.1.617.2 in comparison to the native spike protein (Table S7). Hence, SPR can actively detect the interaction of human recombinant ACE-2 with the various spike proteins immobilized on the SPR sensor.

**Scheme S2.** A) Scheme of the inhibition measurement. The COVID-positive sera (diluted 1:5) are injected on the SPR chip functionalized with the spike protein (native or VOCs). Following a rapid wash in running buffer, the surface was then exposed to 5 µg/mL ACE-2. The SPR signal in RU is expected to range between the positive control (high ACE-2 signal, panel B) and above the negative control signal (low ACE-2 signal, panel C). B) The positive control channel is first exposed to a blank serum at the same dilution factor as the pseudo-neutralization channel and then exposed to ACE-2 in otherwise identical conditions. C) The negative control consist in the injection of the same COVID-positive serum as in A), but running buffer is injected in the second step instead of ACE-2. This provides the background SPR signal and accounts for partial desorption of the antibodies on the SPR sensor.

*Calculation of an inhibition percentage with SPR*

As antibodies binding to spike protein can dissociate from the surface of the SPR chip in running buffer, we added a negative control to the inhibition assay protocol. In one of the four channels, the serum from a COVID-positive individual is first injected in identical conditions to the measurement channels, but running buffer is then injected instead of ACE-2 to measure the drop in SPR response from washing away loosely bound antibodies. The drop in SPR signal from loosely bound antibodies is approximately 50 to 100 RU on average, or about 5% to 10% of the immobilized antibodies (Figure S13). As such, the inhibition protocol required the measurement of the serum samples in duplicate, a positive control and a negative control, as shown in Table S8.

Equation S1 led to the calculation of percent inhibition (%I). As the serum was diluted for the inhibition assay, it should not be interpreted as an absolute value.

(S1) %I = (SPR_COVID+_ – SPR_pos_) / (SPR_pos_ – SPR_neg_) * 100%

Where SPR_COVID+_ is the SPR response (all in RU) of human recombinant ACE-2 following the injection of a sera from a COVID-positive individual, SPR_pos_ corresponds to the SPR response for the human recombinant ACE-2−spike protein interaction in a control serum (devoid of anti-spike antibodies) and SPR_neg_ is the SPR response for the injection of the immune serum and a blank (no human recombinant ACE-2). A percentage change of the inhibition (Δ%I) was calculated from the inhibition with the VOC spike proteins (%I_VOC_) and the inhibition with the native spike protein (%I_native_) (equation 2).

(S2) Δ%I = %I_VOC_ / %I_native_ * 100%

*ELISA ACE-2:spike inhibition assay*

To correlate the neutralization results obtained in SPR, we performed an ELISA inhibition assay inspired by Abe's protocol (*2*) using GenScript SARS-CoV-2 Neutralizing Antibody Standard. The spike antigen (diluted to 2.5 μg/mL in PBS) was first adsorbed to 96-well clear Immulon 4 HBX plates (Thermo Fisher Scientific, 3855) in PBS overnight at 4°C and then washed 3 times with 200 μL PBS plus 0.1% Tween-20 (PBS-T). Plates were blocked for 1 hour at room temperature with 200 μL PBS-T containing 3% w/v milk powder and washed 3 times with 200 μL PBS-T. Patient samples (1:10) and SARS-CoV-2 Neutralizing Antibody Standard (GenScript, A02087) (6000 – 93.75 U/mL) diluted in PBS-T containing 1% w/v milk powder were then added to the plates and incubated for 1 hour at room temperature (100 μL total volume) in triplicates. Positive (calibrator, NIBSC, 20/162) and negative (pre-COVID serum) controls were also added to each plate. Wells were washed 3 times with 200 μL PBS-T and a biotinylated ACE-2 protein (Sigma, SAE0171) was added at 500 ng/mL for 1 hour at room temperature. Wells were washed 3 times with 200 μL PBS-T and 100 μL of a streptavidin-HRP conjugate solution (Sigma, S2438) diluted at 100µg/mL was added for 1 hour at room temperature. After the same washing procedure, a volume of 100 µL TMB-ELISA Substrate Solution (ESBE Scientific, SCY‑TM4999) was added for 20 minutes. The reaction was stopped with 100 μL stop solution containing 1N sulfuric acid (ThermoFisher Scientific, AC124240010). The plates were read in a spectrophotometer (BioTek Instruments Inc., Synergy H1) at 450/630 nm.

The optical densities of the working standard were used to calculate the percentages of inhibition according to the following formula:

(S3) % Inhibition = (1-(OD value of sample/OD value of background))*100%

The resulting linear regression was used to estimate percent inhibition of the patient samples.

**Table S1.** Compilation of the ELISA OD_450_ for the detection of anti-spike antibodies to the spike protein of native SARS-CoV-2 and of the B.1.351 VOC.

|  |  |  | Native Spike | | B.1.351 (SA) Spike | |
| --- | --- | --- | --- | --- | --- | --- |
| Sample ID | **Sex** | **Control PCR test result** | Week 4 | Week 16 | Week 4 | Week 16 |
| 4908 | F | Negative | 1.985 | 1.077 | 1.160 | 0.706 |
| 4910 | M | negative | 2.376 | 1.952 | 1.566 | 1.473 |
| 4913 | F | Negative | 1.628 | 1.261 | 1.123 | 0.694 |
| 4914 | M | Positive | 1.191 | 0.997 | 0.420 | 0.562 |
| 4915 | M | Positive | 1.711 | 1.367 | 1.077 | 0.643 |
| 4916 | F | Positive | 1.718 | 1.044 | 1.243 | 0.729 |
| 4917 | F | Positive | 0.745 | 0.756 | 0.333 | 0.383 |
| 4918 | F | Positive | 1.494 | 1.061 | 0.791 | 0.784 |
| 5902 | F | Negative | 1.981 | 1.262 | 1.450 | 1.283 |
| 5903 | M | Negative | 2.845 | 1.556 | 1.953 | 1.135 |
| 5908 | M | Positive | 3.341 | 2.728 | 2.283 | 2.179 |
| 5909 | M | Positive | 0.960 | 0.887 | 0.590 | 0.576 |
| 5911 | F | Positive | 2.537 | 1.739 | 1.935 | 1.061 |
| 5912 | M | Positive | 2.899 | 2.042 | 2.249 | 1.393 |
| 5914 | F | Positive | 0.752 | 1.663 | 0.525 | 1.061 |
| 5915 | F | Positive | 1.189 | 0.738 | 0.900 | 0.756 |
| 6903 | M | Positive | 2.880 | N/A | 2.059 | N/A |
| 6905 | F | Positive | 3.413 | 2.002 | 2.305 | 3.061 |
| 6906 | F | Positive | 1.409 | 1.134 | 0.870 | 1.351 |
| 6907 | F | Negative | 2.800 | 2.417 | 1.508 | 2.286 |
| 6908 | F | Positive | 1.944 | 1.526 | 1.117 | 0.810 |
| 6909 | M | Positive | 1.821 | 1.080 | 0.690 | 0.743 |
| 6910 | M | Positive | 1.713 | 1.288 | 1.019 | 1.701 |
| 6911 | M | Positive | 2.091 | 1.139 | 0.968 | 2.089 |
| 7003 | F | Negative | 2.342 | 1.844 | 2.735 | 2.782 |
| 7004 | M | Positive | 2.779 | 2.249 | 2.289 | 2.973 |
| 7005 | F | Positive | 2.341 | 2.049 | 1.751 | 2.022 |
| 7006 | M | Positive | 2.313 | 2.408 | 2.270 | 2.360 |
| 7007 | M | Positive | 2.058 | 2.386 | 2.292 | 2.920 |
| 7008 | F | Positive | 2.049 | 2.438 | 2.249 | 2.173 |
| 7009 | F | Positive | 1.853 | 2.024 | 1.475 | 2.407 |
| 7010 | F | Positive | 1.354 | 1.460 | 1.311 | 2.549 |
| C001 | F | ND | 0.155 | 0.33 | 0.125 | 0.133 |
| C002 | F | ND | 0.170 | 0.300 | 0.126 | 0.162 |
| C003 | F | ND | 0.228 | 0.329 | 0.236 | 0.154 |
| C004 | M | ND | 0.268 | 0.281 | 0.147 | 0.185 |
| C005 | F | ND | 0.140 | 0.381 | 0.128 | 0.152 |
| C007 | F | ND | 0.149 | 0.171 | 0.139 | 0.300 |
| C008 | F | ND | 0.162 | 0.247 | 0.167 | 0.139 |
| C009 | F | ND | 0.194 | 0.549 | 0.176 | 0.139 |

N/A: sample not available

ND: not done

**Table S2.** Compilation of the ELISA OD_450_ for the detection of anti-spike antibodies to the spike proteins of B.1.617.2 and P.1 SARS-CoV-2 VOCs.

|  |  |  | B.1.617.2 Spike | | P.1 Spike | |
| --- | --- | --- | --- | --- | --- | --- |
| Sample ID | **Sex** | **Control PCR test result** | Week 4 | Week 16 | Week 4 | Week 16 |
| 4908 | F | Negative | 1.280 | 0.948 | 1.259 | 0.681 |
| 4910 | M | Negative | 1.438 | 1.206 | 1.845 | 1.208 |
| 4913 | F | Negative | 1.342 | 0.896 | 1.144 | 0.730 |
| 4914 | M | Positive | 0.869 | 0.55 | 0.804 | 0.543 |
| 4915 | M | Positive | 1.151 | 0.869 | 1.911 | 0.832 |
| 4916 | F | Positive | 1.214 | 0.976 | 1.250 | 0.606 |
| 4917 | F | Positive | 0.476 | 0.334 | 0.762 | 0.343 |
| 4918 | F | Positive | 1.069 | 1.089 | 1.068 | 0.914 |
| 5902 | F | Negative | 1.502 | 1.091 | 1.676 | 1.679 |
| 5903 | M | Negative | N/A | 1.055 | N/A | 1.590 |
| 5908 | M | Positive | 2.21 | 1.579 | 2.139 | 2.432 |
| 5909 | M | Positive | 0.608 | 0.601 | 0.832 | 0.656 |
| 5911 | F | Positive | 1.726 | 1.295 | 2.191 | 1.349 |
| 5912 | M | Positive | 2.069 | 1.649 | 2.081 | 1.952 |
| 5914 | F | Positive | 0.944 | 0.363 | 1.549 | 1.379 |
| 5915 | F | Positive | 1.055 | 0.971 | 1.015 | 1.186 |
| 6903 | M | Positive | N/A | N/A | N/A | N/A |
| 6905 | F | Positive | 1.947 | 1.672 | 1.606 | 1.883 |
| 6906 | F | Positive | 0.665 | 0.522 | 0.701 | 0.849 |
| 6907 | F | Negative | 1.672 | 1.088 | 1.483 | 1.371 |
| 6908 | F | Positive | 1.067 | 0.753 | 0.914 | 0.767 |
| 6909 | M | Positive | 0.991 | 0.679 | 0.768 | 0.539 |
| 6910 | M | Positive | 0.910 | 0.901 | 0.702 | 0.854 |
| 6911 | M | Positive | 1.316 | 0.9 | 0.876 | 0.992 |
| 7003 | F | Negative | 2.383 | 1.577 | 2.069 | 1.494 |
| 7004 | M | Positive | 1.956 | 1.002 | 1.771 | 1.102 |
| 7005 | F | Positive | 1.907 | 1.073 | 1.659 | 1.315 |
| 7006 | M | Positive | 2.017 | 1.225 | 1.704 | 1.231 |
| 7007 | M | Positive | 2.44 | 1.113 | 2.241 | 1.337 |
| 7008 | F | Positive | 2.228 | 1.29 | 2.244 | 1.389 |
| 7009 | F | Positive | 1.627 | 0.824 | 1.567 | 1.020 |
| 7010 | F | Positive | 1.215 | 0.723 | 1.550 | 1.061 |
| C001 | F | ND | 0.014 |  |  |  |
| C002 | F | ND | 0.030 |  |  |  |
| C003 | F | ND | 0.002 |  |  |  |
| C004 | M | ND | 0.083 |  |  |  |
| C005 | F | ND | 0.003 |  |  |  |
| C007 | F | ND | 0.003 |  |  |  |
| C008 | F | ND | 0.021 |  |  |  |
| C009 | F | ND | -0.009 |  |  |  |

N/A: sample not available

ND: not done

Controls were performed once for all sets of measurements

**Table S3.** Compilation of the SPR binding shift for the detection of anti-spike antibodies to the spike protein of native SARS-CoV-2 and of the B.1.351 VOC. All shifts are reported in RU for the secondary detection step.

|  |  |  | Native Spike | | B.1.351 (SA) Spike | |
| --- | --- | --- | --- | --- | --- | --- |
| Sample ID | **Sex** | **Control PCR test result** | Week 4 | Week 16 | Week 4 | Week 16 |
| 4908 | F | Negative | 1181 | 1075 | 880 | 360 |
| 4910 | M | Negative | 1862 | 1572 | 797 | 104 |
| 4913 | F | Negative | 1278 | 933 | 803 | 1807 |
| 4914 | M | Positive | 1124 | 1475 | 717 | 400 |
| 4915 | M | Positive | 1885 | 1844 | 785 | 1807 |
| 4916 | F | Positive | 313 | 1113 | 1036 | 875 |
| 4917 | F | Positive | 1436 | 601 | 453 | 1212 |
| 4918 | F | Positive | 1539 | 773 | 846 | 792 |
| 5902 | F | Negative | 2341 | 1726 | 422 | 1132 |
| 5903 | M | Negative | 1968 | 1990 | 1725 | 1205 |
| 5908 | M | Positive | 1978 | 1977 | 691 | 1742 |
| 5909 | M | Positive | 1342 | 775 | 1061 | 517 |
| 5911 | F | Positive | 1531 | 2177 | 1722 | 1617 |
| 5912 | M | Positive | 2024 | 2283 | 933 | 1704 |
| 5914 | F | Positive | 1298 | 1858 | 1608 | 1071 |
| 5915 | F | Positive | 1621 | 121 | 1070 | 1063 |
| 6903 | M | Positive | 1996 | N/A | 1513 | N/A |
| 6905 | F | Positive | 897 | 2188 | 1013 | 1566 |
| 6906 | F | Positive | 910 | 733 | 1825 | 331 |
| 6907 | F | Negative | 687 | 1202 | 438 | 915 |
| 6908 | F | Positive | 1545 | 1310 | 79 | 1108 |
| 6909 | M | Positive | 1047 | 444 | 1039 | 593 |
| 6910 | M | Positive | 713 | 1304 | 1087 | 779 |
| 6911 | M | Positive | 1686 | 1579 | 1558 | 726 |
| 7003 | F | Negative | 2662 | 1974 | 2089 | 1110 |
| 7004 | M | Positive | 1988 | 1534 | 2118 | 937 |
| 7005 | F | Positive | 1101 | 1661 | 1275 | 160 |
| 7006 | M | Positive | 1095 | 1773 | 462 | 1362 |
| 7007 | M | Positive | 908 | 1891 | 1868 | 1092 |
| 7008 | F | Positive | 1603 | 1312 | 1593 | 42 |
| 7009 | F | Positive | 1744 | 1836 | 1445 | 1210 |
| 7010 | F | Positive | 1346 | 1424 | 870 | 757 |
| C001 | F | ND | 280 | -47 | 386 | 98 |
| C002 | F | ND | 3 | 240 | 412 | 183 |
| C003 | F | ND | 349 | 108 | 454 | 158 |
| C004 | M | ND | 261 | 94 | 672 | 158 |
| C005 | F | ND | 271 | 24 | 677 | 343 |
| C007 | F | ND | 441 | 127 | 622 | 119 |
| C008 | F | ND | 582 | 6 | 1276 | 51 |
| C009 | F | ND | 217 | 307 | 622 | 277 |

N/A: sample not available

ND: not done

**Table S4.** Compilation of the SPR binding shift for the detection of anti-spike antibodies to the spike proteins of B.1.617.2 and P.1 SARS-CoV-2 VOCs. All shifts are reported in RU for the secondary detection step.

|  |  |  | B.1.617.2 Spike | | P.1 Spike | |
| --- | --- | --- | --- | --- | --- | --- |
| Sample ID | **Sex** | **Control PCR test result** | Week 4 | Week 16 | Week 4 | Week 16 |
| 4908 | F | Negative | 1762 | 498 | 697 | 706 |
| 4910 | M | Negative | 1127 | 686 | 617 | 1126 |
| 4913 | F | Negative | 1008 | 945 | 852 | 428 |
| 4914 | M | Positive | 7 | 30 | 349 | 293 |
| 4915 | M | Positive | 227 | 1412 | 767 | 603 |
| 4916 | F | Positive | 134 | 169 | 668 | 544 |
| 4917 | F | Positive | 493 | 583 | 204 | 163 |
| 4918 | F | Positive | 319 | 77 | 844 | 603 |
| 5902 | F | Negative | 212 | 379 | 1424 | 685 |
| 5903 | M | Negative | N/A | 1187 | 976 | 1016 |
| 5908 | M | Positive | 1494 | 487 | 168 | 1065 |
| 5909 | M | Positive | 899 | 351 | 469 | 191 |
| 5911 | F | Positive | 2595 | 522 | 1555 | 882 |
| 5912 | M | Positive | 920 | 1713 | 1311 | 628 |
| 5914 | F | Positive | 1404 | 983 | 1091 | 911 |
| 5915 | F | Positive | 153 | 1319 | 732 | 895 |
| 6903 | M | Positive | 1158 | N/A | 1062 | N/A |
| 6905 | F | Positive | 1268 | 580 | 1119 | 1050 |
| 6906 | F | Positive | 1312 | 764 | 512 | 465 |
| 6907 | F | Negative | 2150 | 1613 | 1080 | 1132 |
| 6908 | F | Positive | 1559 | 1101 | 667 | 593 |
| 6909 | M | Positive | 146 | 1683 | 747 | 629 |
| 6910 | M | Positive | 1326 | 1132 | 786 | 462 |
| 6911 | M | Positive | 1438 | 1058 | 880 | 877 |
| 7003 | F | Negative | 1837 | 400 | 958 | 940 |
| 7004 | M | Positive | 498 | 1587 | 878 | 1169 |
| 7005 | F | Positive | 1606 | 1405 | 1617 | 268 |
| 7006 | M | Positive | 1836 | 107 | 1112 | 405 |
| 7007 | M | Positive | 1648 | 1322 | 1216 | 782 |
| 7008 | F | Positive | 1530 | 1092 | 1083 | 1328 |
| 7009 | F | Positive | 1981 | 1805 | 945 | 836 |
| 7010 | F | Positive | 900 | 1901 | 947 | 606 |
| C001 | F | ND | 220 | 220 | 239 | 244 |
| C002 | F | ND | 824 | 824 | 461 | 158 |
| C003 | F | ND | 35 | 35 | 226 | 347 |
| C004 | M | ND | 637 | 637 | 601 | 627 |
| C005 | F | ND | 122.5 | 122.5 | 591 | 604 |
| C007 | F | ND | 261.5 | 261.5 | 528 | 841 |
| C008 | F | ND | 87 | 87 | 59 | 420 |
| C009 | F | ND | 110.2 | 110.2 | N/A | N/A |

N/A: sample not available

ND: not done

**Table S5.** Compilation of the ELISA OD_450_ for the detection of anti-RBD antibodies to the spike protein of native SARS-CoV-2 and of the B.1.351 VOC.

|  |  |  | Native RBD | | B.1.351 (SA) RBD | |
| --- | --- | --- | --- | --- | --- | --- |
| Sample ID | **Sex** | **Control PCR test result** | Week 4 | Week 16 | Week 4 | Week 16 |
| 4908 | F | Negative | 0.155 | 0.136 | 0.769 | 0.168 |
| 4910 | M | Negative | 0.165 | 0.341 | 1.220 | 0.672 |
| 4913 | F | Negative | 0.213 | 0.157 | 0.785 | 0.153 |
| 4914 | M | Positive | 0.130 | 0.108 | 0.435 | 0.183 |
| 4915 | M | Positive | 0.203 | 0.218 | 0.890 | 0.364 |
| 4916 | F | Positive | 0.172 | 0.212 | 0.937 | 0.355 |
| 4917 | F | Positive | 0.112 | 0.120 | 0.303 | 0.109 |
| 4918 | F | Positive | 0.143 | 0.145 | 0.687 | 0.152 |
| 5902 | F | Negative | 0.208 | 0.327 | 0.775 | 0.263 |
| 5903 | M | Negative | 0.334 | 0.384 | 1.369 | 0.364 |
| 5908 | M | Positive | 0.564 | 0.592 | 1.554 | 1.121 |
| 5909 | M | Positive | 0.122 | 0.113 | 0.465 | 0.132 |
| 5911 | F | Positive | 0.397 | 0.486 | 1.470 | 0.482 |
| 5912 | M | Positive | 0.537 | 0.369 | 1.487 | 0.771 |
| 5914 | F | Positive | 0.116 | 0.356 | 0.308 | 0.327 |
| 5915 | F | Positive | 0.182 | 0.180 | 0.361 | 0.201 |
| 6903 | M | Positive | 0.348 | N/A | 1.101 | N/A |
| 6905 | F | Positive | 0.268 | 0.474 | 1.022 | 0.204 |
| 6906 | F | Positive | 0.125 | 0.192 | 0.410 | 0.258 |
| 6907 | F | Negative | 0.159 | 0.235 | 0.787 | 0.243 |
| 6908 | F | Positive | 0.218 | 0.148 | 0.617 | 0.144 |
| 6909 | M | Positive | 0.185 | 0.116 | 0.260 | 0.137 |
| 6910 | M | Positive | 0.200 | 0.128 | 0.638 | 0.263 |
| 6911 | M | Positive | 0.144 | 0.145 | 0.588 | 0.139 |
| 7003 | F | Negative | 0.978 | 0.199 | 1.444 | 0.593 |
| 7004 | M | Positive | 0.365 | 0.170 | 1.050 | 0.271 |
| 7005 | F | Positive | 0.373 | 0.161 | 1.019 | 0.276 |
| 7006 | M | Positive | 0.610 | 0.154 | 1.237 | 0.539 |
| 7007 | M | Positive | 0.616 | 0.207 | 1.411 | 0.440 |
| 7008 | F | Positive | 0.555 | 0.237 | 1.216 | 0.388 |
| 7009 | F | Positive | 0.373 | 0.180 | 0.755 | 0.281 |
| 7010 | F | Positive | 0.302 | 0.194 | 0.636 | 0.569 |
| C001 | F | ND | 0.130 | 0.108 | 0.240 | 0.107 |
| C002 | F | ND | 0.103 | 0.101 | 0.115 | 0.105 |
| C003 | F | ND | 0.194 | 0.104 | 0.352 | 0.104 |
| C004 | M | ND | 0.116 | 0.107 | 0.101 | 0.104 |
| C005 | F | ND | 0.114 | 0.111 | 0.114 | 0.100 |
| C007 | F | ND | 0.101 | 0.104 | 0.137 | 0.099 |
| C008 | F | ND | 0.111 | 0.109 | 0.199 | 0.105 |
| C009 | F | ND | 0.117 | 0.110 | 0.162 | 0.113 |

N/A: sample not available

ND: not done

**Table S6.** Compilation of the SPR binding shift for the detection of anti-RBD antibodies for the native SARS-CoV-2 RBD and for the B.1.351 VOCs. All shifts are reported in RU for the secondary detection step.

|  |  |  | Native RBD | | B.1.351 (SA) RBD | |
| --- | --- | --- | --- | --- | --- | --- |
| Sample ID | **Sex** | **Control PCR test result** | Week 4 | Week 16 | Week 4 | Week 16 |
| 4908 | F | Negative | 268 | 173 | -16 | 474 |
| 4910 | M | Negative | 186 | 593 | 32 | 267 |
| 4913 | F | Negative | 101 | 136 | -132 | -63 |
| 4914 | M | Positive | 257 | 417 | 36 | -134 |
| 4915 | M | Positive | 331 | 226 | -2 | 22 |
| 4916 | F | Positive | 338 | 446 | -52 | 16 |
| 4917 | F | Positive | 79 | 266 | -79 | -50 |
| 4918 | F | Positive | 183 | 255 | -127 | -61 |
| 5902 | F | Negative | 558 | 583 | 71 | 5 |
| 5903 | M | Negative | 615 | 474 | 128 | 83 |
| 5908 | M | Positive | 475 | 817 | 711 | -11 |
| 5909 | M | Positive | 235 | 401 | -26 | -72 |
| 5911 | F | Positive | 656 | 622 | 136 | -33 |
| 5912 | M | Positive | 759 | 716 | 224 | 151 |
| 5914 | F | Positive | 87 | 612 | -5 | 32 |
| 5915 | F | Positive | 405 | 494 | 124 | -24 |
| 6903 | M | Positive | 452 | N/A | 47 | N/A |
| 6905 | F | Positive | 228 | 277 | 110 | -198 |
| 6906 | F | Positive | 334 | 502 | 47 | -139 |
| 6907 | F | Negative | 601 | 407 | -79 | -95 |
| 6908 | F | Positive | 88 | 446 | 48 | -148 |
| 6909 | M | Positive | 181 | 530 | -10 | -63 |
| 6910 | M | Positive | 327 | 435 | -33 | -177 |
| 6911 | M | Positive | 444 | 610 | -102 | -17 |
| 7003 | F | Negative | 184 | 810 | 267 | -22 |
| 7004 | M | Positive | 512 | 1040 | 151 | 262 |
| 7005 | F | Positive | 527 | 1144 | 87 | 30 |
| 7006 | M | Positive | 562 | 910 | 108 | 190 |
| 7007 | M | Positive | 933 | 777 | 379 | 209 |
| 7008 | F | Positive | 592 | 657 | 80 | 68 |
| 7009 | F | Positive | 719 | 685 | -5 | -28 |
| 7010 | F | Positive | 797 | 397 | 99 | 241 |
| C001 | F | ND | 187 | 257 | 22 | -31 |
| C002 | F | ND | 268 | 222 | -43 | 70 |
| C003 | F | ND | 36 | 337 | -22 | -52 |
| C004 | M | ND | 252 | 273 | -99 | -81 |
| C005 | F | ND | 216 | 314 | 45 | 11 |
| C007 | F | ND | 310 | 351 | -45 | 75 |
| C008 | F | ND | 127 | 304 | -43 | -52 |
| C009 | F | ND | 177 | 273 | -97 | -162 |

N/A: sample not available

ND: not done

**Table S7.** ELISA for the detection of IgG targeting the native and B.1.351 RBD proteins

|  |  | Native RBD | | B.1.351 RBD | |
| --- | --- | --- | --- | --- | --- |
| ELISA |  | Week 4 | Week 16 | Week 4 | Week 16 |
| OD_450_ (A.U) | Pos. | 0.3 ± 0.2 | 0.23 ± 0.12 | 0.9 ± 0.4 | 0.3 ± 0.2 |
| OD_450_ (A.U) | Neg. | 0.12 ± 0.03 | 0.107 ± 0.003 | 0.18 ± 0.08 | 0.105 ± 0.004 |
| Threshold (A.U) |  | 0.13 | 0.125 | 0.25 | 0.125 |
| COVID+ sera | # Pos. | 27 | 27 | 32 | 30 |
|  | # Neg. | 5 | 4 | 0 | 1 |
| Control sera | # Pos. | 1 | 0 | 1 | 0 |
|  | # Neg. | 7 | 8 | 8 | 8 |
| AUC |  | 0.91 | 0.99 | 0.99 | 0.99 |
| p value |  | <0.05 | <0.01 | <0.0001 | <0.01 |
| Sensitivity |  | 84 | 87 | 100 | 97 |
| Specificity |  | 87 | 100 | 88 | 100 |

**Table S8.** SPR assay for the detection of IgG targeting the native and B.1.351 RBD proteins

|  |  | Native RBD | | B.1.351 RBD | |
| --- | --- | --- | --- | --- | --- |
|  |  | Week 4 | Week 16 | Week 4 | Week 16 |
| Shift (kRU) | Pos. | 0.4 ± 0.2 | 0.5 ± 0.2 | 0.07 ± 0.16 | 0.02 ± 0.15 |
| Shift (kRU) | Neg. | 0.18 ± 0.08 | 0.29 ± 0.04 | -0.04 ± 0.05 | -0.03 ± 0.08 |
| Threshold (kRU) |  | 0.3 | 0.4 | 0.05 | 0.1 |
| COVID+ sera | # Pos. | 20 | 24 | 13 | 7 |
|  | # Neg. | 12 | 7 | 19 | 24 |
| Control sera | # Pos. | 1 | 0 | 0 | 0 |
|  | # Neg. | 7 | 8 | 8 | 8 |
| AUC |  | 0.77 | 0.84 | 0.77 | 0.58 |
| p value |  | <0.05 | <0.01 | 0.08 | 0.37 |
| Sensitivity |  | 62 | 77 | 41 | 23 |
| Specificity |  | 88 | 100 | 100 | 100 |

**Table S9.** Spike protein / ACE-2 binding kinetics and affinity

| SARS-CoV-2 strain | k_on_ (M^-1.^s^-1^) x 10^4^ | k_off_ (s^-1^) x 10^-4^ | K_D_ (nM) |
| --- | --- | --- | --- |
| native | 6.7 ± 1.0 | 25.2 ± 4.4 | 37.6 ± 1.2 |
| B.1.351 | 3.6 ± 0.5 | 11.9 ± 1.6 | 32.9 ± 1.6 |
| P.1 | 4.2 ± 1.0 | 13 ± 5 | 31 ± 6 |
| B.1.617.2 | 5.3 ± 0.3 | 19.9 ± 0.4 | 37.7 ± 1.6 |

**Table S10.** Protocol / injection sequence for the SPR inhibition assay

|  | **Channel A** | **Channel B** | **Channel C** | **Channel D** |
| --- | --- | --- | --- | --- |
| **Step time (min)** | Measurement | Measurement | Positive control | Negative control |
| **10** | COVID+ serum | COVID+ serum | Negative serum | COVID+ serum |
| **Few seconds** | Running buffer | Running buffer | Running buffer | Running buffer |
| **10** | 5 µg/mL ACE-2 | 5 µg/mL ACE-2 | 5 µg/mL ACE-2 | Running buffer |
| **Few seconds** | Regeneration | Regeneration | Regeneration | Regeneration |

**Table S11.** Compilation of the ELISA and SPR inhibition data (expressed in % inhibition) for the native spike protein and for the B.1.351, B.1.617.1, B.1.617.2, and P.1 VOCs using serum from SARS-CoV-2-positive individuals at week 4 and 16 post-diagnosis.

|  |  | NATIVE | NATIVE | | B.1.351 | | B.1.617.1 | | B.1.617.2 | | P.1 | |
| --- | --- | --- | --- | --- | --- | --- | --- | --- | --- | --- | --- | --- |
| Sample ID | **Sex** | **W4 ELISA** | **W4** | **W16** | **W4** | **W16** | **W4** | **W16** | **W4** | **W16** | **W4** | **W16** |
| 4908 | F | 26 | 57 | 31 | 23 | 25 | 10 | -14 | -5 | 3 | 44 | 18 |
| 4910 | M | N/A | 47 | 51 | 25 | 48 | 8 | 5 | -33 | 10 | 58 | -1 |
| 4913 | F | 25 | 23 | 73 | 28 | 26 | 1 | -22 | -23 | 6 | 26 | 22 |
| 4914 | M | 25 | 34 | 34 | 18 | 32 | -11 | -15 | -20 | 7 | 12 | 11 |
| 4915 | M | 31 | 33 | 78 | 28 | 30 | 10 | 6 | 5 | 11 | 37 | 19 |
| 4916 | F | 32 | 38 | 15 | 16 | 16 | 4 | -9 | 1 | 8 | 15 | 14 |
| 4917 | F | 20 | 36 | 35 | 23 | 36 | 1 | -3 | -4 | 6 | 30 | 20 |
| 4918 | F | 27 | 38 | 56 | 27 | 39 | 8 | -5 | 38 | 14 | 11 | 17 |
| 5902 | F | 31 | 56 | 42 | 41 | 47 | 16 | 15 | 38 | 40 | 36 | 39 |
| 5903 | M | 55 | 82 | 76 | 57 | 32 | 50 | 0 | 108 | 98 | N/A | 19 |
| 5908 | M | 85 | 92 | 97 | 92 | 52 | 78 | 81 | 27 | 19 | 83 | 71 |
| 5909 | M | 21 | 40 | 31 | 55 | 46 | 24 | 14 | 51 | 33 | 21 | 19 |
| 5911 | F | 58 | 69 | 65 | 52 | 33 | 37 | 26 | 97 | 52 | 45 | 35 |
| 5912 | M | 86 | 90 | 78 | 89 | 42 | 69 | 45 | 54 | 31 | 86 | 69 |
| 5914 | F | 18 | 46 | 52 | 14 | 23 | 23 | 37 | 33 | 32 | 24 | 6 |
| 5915 | F | 26 | 54 | 48 | 10 | 15 | 27 | 26 | 43 | 27 | 0 | 7 |
| 6903 | M | 55 | 56 | N/A | 19 | N/A | 40 | N/A | 64 | N/A | 47 | N/A |
| 6905 | F | 62 | 70 | 68 | 86 | 78 | 44 | 57 | 81 | 40 | 62 | 54 |
| 6906 | F | 19 | 31 | 42 | 8 | 35 | -6 | 49 | 19 | 36 | 38 | 13 |
| 6907 | F | 51 | 81 | 107 | 37 | 27 | 77 | 42 | 71 | 68 | 36 | 16 |
| 6908 | F | 29 | 51 | 16 | 33 | 57 | 19 | 27 | 41 | 31 | 40 | 53 |
| 6909 | M | 24 | 8 | 19 | 3 | 16 | 12 | 25 | 27 | 21 | 28 | 27 |
| 6910 | M | 44 | 5 | 48 | 34 | 41 | 13 | 24 | 25 | 27 | 21 | 44 |
| 6911 | M | 42 | 73 | 68 | 54 | 33 | 12 | 27 | 14 | 7 | 33 | 11 |
| 7003 | F | 76 | 87 | 68 | 74 | 24 | 65 | 26 | 66 | 48 | 63 | 41 |
| 7004 | M | 74 | 90 | 92 | 78 | 56 | 99 | 52 | 81 | 76 | 70 | 70 |
| 7005 | F | 70 | 41 | 82 | 31 | 10 | 59 | 31 | 57 | 35 | 53 | 42 |
| 7006 | M | 81 | 91 | 77 | 40 | 37 | 86 | 52 | 75 | 66 | 66 | 47 |
| 7007 | M | 83 | 120 | 42 | 64 | 58 | 76 | 45 | 82 | 46 | 67 | 52 |
| 7008 | F | 82 | 103 | 53 | 64 | 45 | 78 | 39 | 77 | 87 | 66 | 18 |
| 7009 | F | 53 | 67 | 96 | 46 | 14 | 58 | 31 | 55 | 41 | 13 | 21 |
| 7010 | F | 36 | 72 | 13 | 53 | 41 | 55 | 36 | 55 | 39 | 37 | 48 |
| Mean |  |  | 59 | 57 | 41 | 36 | 36 | 24 | 41 | 34 | 41 | 30 |
| σ |  |  | 28 | 26 | 25 | 15 | 31 | 24 | 36 | 25 | 22 | 20 |

N/A: sample not available

**Table S12.** Compilation of the SPR inhibition data for the native and B.1.617.2 SARS-CoV-2 spike proteins using serum from vaccinated individuals.

| Sample ID | Sex | Vaccine | Weeks post diagnosis | Weeks post vaccine | NATIVE | B.1.617.2 |
| --- | --- | --- | --- | --- | --- | --- |
| 4910 | M | BNT162b2 | 24 | 4 | 97 | 76 |
| 4932 | F | BNT162b2 | 24 | 16 | 90 | 84 |
| 4933 | F | BNT162b2 | 24 | 8 | 85 | 99 |
| 5914 | F | BNT162b2 | 24 | 5 | 96 | 89 |
| 5916 | F | BNT162b2 | 24 | 8 | 96 | 95 |
| 6912 | F | BNT162b2 | 24 | 6 | 99 | 85 |
| 6915 | F | BNT162b2 | 24 | 2 | 94 | 103 |
| 6916 | M | ChAdOx1 nCoV-19 | 24 | 11 | 106 | 89 |
| 7016 | F | BNT162b2 | 16 | 4 | 103 | 100 |
| Mean |  |  |  |  | 96 | 91 |
| σ |  |  |  |  | 6 | 9 |

**Figure S1**. ELISA OD_450_ (top) and SPR binding shifts (bottom) for the detection of anti-spike IgG in each positive serum (n=32) and negative controls (n=8) at week 4 (W4) and week 16 (W16) post-infection for the native and B.1.351, B.1.617.2, and P.1 spike proteins. As shown in Figure 1, the average ELISA OD and the SPR binding shift was generally lower for the VOCs, with the P.1 leading to the lowest cross-reactivity of the IgG antibodies elicited to the native SARS-CoV-2. In all cases, the controls associated to an experiment are represented to the left of the data for the positive sera. * p< 0.05, ** p< 0.01, *** p < 0.001, **** p < 0.0001

**Figure S2**. ELISA OD_450_ for the detection of anti-spike IgG in SARS-CoV-2 positive serum at week 4 (W4) and week 16 (W16) post-diagnosis for the native and B.1.351 spike proteins. Among these positive individuals, antibody OD_450_ did not differ (p values ranged from 0.26 to 0.90) between individuals that recovered (Neg, n=7) from the ones that did not fully recovered (Pos, n = 25). Recovery was determined with a second PCR test at the time of enrollment.

**Figure S3**. Receiver-operator characteristic curves (ROC) for the SPR detection of anti-IgG targeting the spike proteins (native, B.1.351, B.1.617.2, and P.1) in serum samples of SARS-CoV-2 positive (n=32) and negative (n=8) individuals at weeks 4 and 16 post-infection.

**Figure S4**. Receiver-operator characteristic curves (ROC) for the ELISA detection of anti-IgG targeting the spike proteins (native, B.1.351, B.1.617.2, and P.1) in serum samples of SARS-CoV-2 positive (n=32) and negative (n=8) individuals at weeks 4 and 16 post-infection.

**Figure S5**. Average ELISA OD_450_ (left) and SPR binding shifts (right) for the detection of anti-RBD IgG in positive sera at week 4 (W4, n = 32) and week 16 (W16, n = 31), and negative controls (n=8) post-infection for the native and B.1.351 RBD proteins. In general, poorer contrast was observed with RBD compared to the spike proteins (Figure 1). In all cases, the controls associated to an experiment are represented to the left of the data for the positive sera. ELISA was performed at 1:50 dilution of sera, while SPR required sera to be diluted 1:5.

**Figure S6.** Receiver-operator characteristic curves (ROC) for the ELISA detection of anti-IgG targeting the RBD proteins (native and B.1.351) in serum samples of SARS-CoV-2 positive (n=32 for week 4 and n=31 for week 16) and negative (n=8) individuals at weeks 4 and 16 post-infection.

**Figure S7.** Receiver-operator characteristic curves (ROC) for the SPR detection of anti-IgG targeting the RBD proteins (native and B.1.351) in serum samples of SARS-CoV-2 positive (n=32 for week 4 and n=31 for week 16) and negative (n=8) individuals at weeks 4 and 16 post-infection.

**Figure S8.** Left) Raw data for the SPR sensorgram for the anti-spike protein affinity assay using a SARS-CoV-2-positive serum at different dilution factors. 1 nm is approximately 330 RU. Right) A 1:1 binding site Langmuir isotherm was applied to estimate the K_D_, expressed in units of dilution factor (fractional numbers are used in the x-axis, *i.e.,* 1:5 dilution is 0.2). All data were corrected by the SPR response in the reference channel to remove the contribution of the nonspecific response of the serum.

**Figure S9.** Left) SPR response from a SARS-CoV-2-positive serum as a function of the dilution factor of the serum in running buffer. Right) Decrease in the SPR response from human recombinant ACE-2 following the exposure of the SPR sensor to a SARS-CoV-2-positive serum. The decrease was calculated from the comparison of ACE-2 binding to the spike-coated SPR chip in the absence of SARS-CoV-2-positive serum in otherwise identical conditions. In both panels, the dilution is presented as a fractional number, *i.e*. 1:5 dilution is 0.2.

**Figure S10.** Typical sensorgram for the calibration of human recombinant ACE-2 on a spike SPR surface. Spike was immobilized at 20 µg/mL, while different concentrations of human recombinant ACE-2 were injected sequentially. All human recombinant ACE-2 concentrations are in µg/mL.

**Figure S11.** SPR sensorgram for the detection of ACE-2 for the determination of pseudo-neutralization. The blue curve refers to the positive control (detection of ACE-2 in absence of neutralizing antibody), the black curves are the ACE-2 binding measurements following exposure of the chip to the serum (duplicate), and the red curve shows the background signal in absence of ACE-2 (but with prior injection of the serum), consisting in the negative control.

**Figure S12.** Percent inhibition measured with SPR for an individual aged 18-49 years as a function of weeks following a positive diagnosis. This individual received two doses of the BNT162b2 vaccine on weeks 5 and 20.

**References**

1. F. Tian, B. Tong, L. Sun, S. Shi, B. Zheng, Z. Wang, X. Dong, P. Zheng, Mutation N501Y in RBD of Spike Protein Strengthens the Interaction between COVID-19 and its Receptor ACE2. *bioRxiv*, 2021.2002.2014.431117 (2021).

2. K. T. Abe, Z. Li, R. Samson, P. Samavarchi-Tehrani, E. J. Valcourt, H. Wood, P. Budylowski, A. P. Dupuis, II, R. C. Girardin, B. Rathod, J. H. Wang, M. Barrios-Rodiles, K. Colwill, A. J. McGeer, S. Mubareka, J. L. Gommerman, Y. Durocher, M. Ostrowski, K. A. McDonough, M. A. Drebot, S. J. Drews, J. M. Rini, A.-C. Gingras, A simple protein-based surrogate neutralization assay for SARS-CoV-2. *JCI Insight* **5**, (2020).
